# Understanding Continuance Usage of Mobile Learning Applications: The Moderating Role of Habit

**DOI:** 10.3389/fpsyg.2021.736051

**Published:** 2021-11-10

**Authors:** Yi-Ting Wang, Kuan-Yu Lin

**Affiliations:** ^1^Department of Applied Foreign Languages, Ling Tung University, Taichung, Taiwan; ^2^Department of Information Technology, Ling Tung University, Taichung, Taiwan

**Keywords:** M-learning applications, expectation confirmation theory, flow theory, habit, continuance usage intention

## Abstract

This study explored the factors that affect the intention of users to continue using mobile learning (m-learning) applications (apps). The influence of habit on user behavior toward information systems has been extensively discussed in the literature, but its role in the continuance of users when it comes to their usage of apps, especially m-learning apps, has rarely been reported. To obtain a comprehensive understanding of behaviors regarding the use of m-learning apps, this study constructed a theoretical research framework based on expectation confirmation theory and flow theory by considering habit as a moderating variable. Online questionnaires were administered to users of m-learning apps in Taiwan and data were analyzed through a structural equation modeling approach. The results indicated that the intention of users to continue using m-learning apps was influenced by satisfaction, perceived usefulness, and flow experience. Expectation confirmation affected user satisfaction and perceived usefulness. Differences existed in the intention to continue usage between users with strong and weak habits. In addition, perceived usefulness, expectation confirmation, and flow experience had direct and positive effects on satisfaction. The implications of these findings were discussed.

## Introduction

The emergence of communication technology and mobile devices has contributed to rapid developments in mobile applications (apps). Advances in network communication and mobile technology have considerably changed the lives and learning methods of people (Nikou and Economides, 2017; Chao, 2019; Liu et al., 2021). Researchers (Chao, 2019; Alshurideh et al., 2020; Hoi, 2020) have indicated that online learning combined with mobile technology is becoming an essential educational practice for teachers and students. Because of the portability of mobile devices such as smartphones and tablets, their usage by learners has increased considerably. A growing number of mobile learning (m-learning) apps have been developed (Chao, 2019; Alshurideh et al., 2020). Unlike computer-aided learning, m-learning allows users to learn without time and space limitations, which increases their learning intention and learning effectiveness (Chao, 2019; Quesada-Pallares et al., 2019; Wang et al., 2019; Liu et al., 2021).

Research that has examined mobile technology usage behaviors from the perspective of expectation confirmation theory (ECT) (Hsu and Lin, 2015; Li and Fang, 2019; Alshurideh et al., 2020) has indicated that expectation confirmation positively affects the satisfaction of users with and perceived usefulness of mobile technology; thus, expectation confirmation facilitates the continuance usage of such technology. However, ECT emphasizes the exploration of intention to continue using information technology from a utility perspective (e.g., perceived usefulness) rather than a hedonic perspective (e.g., perceived pleasure) (Bhattacherjee, 2001; Dağhan and Akkoyunlu, 2016; Alshurideh et al., 2020). Some studies (Cheng and Yuen, 2018; Fang et al., 2019; Rodríguez-Ardura and Meseguer-Artola, 2019; Yen and Lin, 2020) have suggested that both the usefulness of information systems and hedonic factors should be considered when information technology is applied in a learning context.

Most studies have explored the hedonic experiences of users based on flow theory (Kim and Hall, 2019; Lee et al., 2020; Yen and Lin, 2020), according to which flow is a pleasant experience of complete absorption in a task. The study of Alraimi et al. (2015) argued that the pleasant feelings of users help to strengthen their motivation to use and continue to use information technology for learning. Researchers (Stavrou et al., 2015; Huang et al., 2017; Rodríguez-Ardura and Meseguer-Artola, 2019; Yen and Lin, 2020) have found that flow experience originates from a balance between challenge and the ability of an individual to overcome the challenge. Thus, flow occurs when an individual has confidence in their ability to overcome a challenge.

The influence of habit on the usage behavior of individuals toward information systems has been widely investigated (Lin et al., 2017; Nascimento et al., 2018; Bolen, 2020). Researchers have argued that users might be reluctant to use information systems simply because of their habits (Lin et al., 2017). Thus, the effect of habits on the behavioral intention of users toward information technology should not be ignored when examining the continuance usage of new technology.

Based on the above-mentioned studies, this study adopted the utility viewpoint and considered positive experiences to examine the adoption of users of information technology for learning. This study also investigated the effect of habit on the intention of learners to continue using such technology. Based on the literature, this study constructed an integrated research model by adopting the utility viewpoint from ECT, the hedonic viewpoint from flow theory, and habit to identify the factors that affect the continuance intention of users to use m-learning apps.

## Research Model and Hypotheses

**FIGURE 1:**
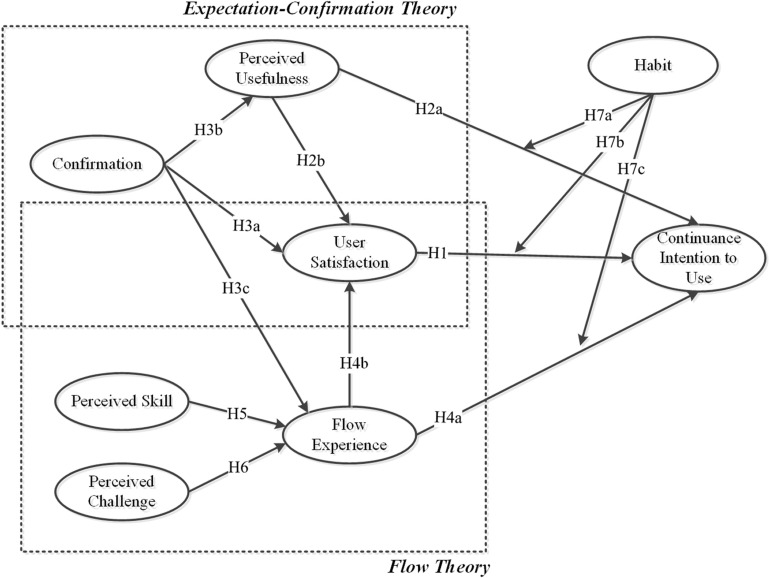
Research model.

Figure 1 displays the research framework for examining continuance intention to use m-learning apps. In this model, expectation confirmation factors (i.e., confirmation, perceived usefulness, and satisfaction), flow characteristics (i.e., perceived skill and perceived challenge), and habit were considered to influence the continuance intention of users to use m-learning apps. The definitions of the constructs used in this study and the research hypotheses were presented in the following text.

### Expectation Confirmation Theory

The study of Oliver (1980) proposed ECT for the field of marketing by extending his satisfaction recognition model. The work of Bhattacherjee (2001) contended that whether a user continues to use an information system is a decision similar to the repurchase-related decision-making behavior of consumers. Therefore, he extended ECT to the information management domain and proposed the information systems continuance model. Research has indicated that user satisfaction and perceived usefulness in using an information system affect continuance usage intention. In addition, user confirmation and perceived usefulness positively affect user satisfaction. User confirmation also affects the perceived usefulness of users of a system.

Numerous studies (Bhattacherjee, 2001; Cheng and Yuen, 2018; Li and Fang, 2019; Alshurideh et al., 2020) had used ECT-based frameworks and concluded that confirmation, perceived usefulness, and user satisfaction were crucial factors affecting the usage behavior of individuals toward information systems. The study of Alshurideh et al. (2020) found that the degree of confirmation is a fundamental factor affecting the satisfaction of users with and behavioral intention toward m-learning systems. The work of Li and Fang (2019) suggested that the degree of confirmation positively affects satisfaction and perceived usefulness, that perceived usefulness significantly affects user satisfaction, and that user satisfaction strongly affects mobile technology usage intention. Therefore, this study argued that the perceptions of users when it comes to the confirmation in and perceived usefulness of using m-learning apps could affect their satisfaction and continuance usage intention. Consequently, the following hypotheses were proposed:

*H1. User satisfaction positively affects continuance intention to use.*

*H2a. Perceived usefulness positively affects continuance intention to use.*

*H2b. Perceived usefulness positively affects user satisfaction.*

*H3a. The degree of the confirmation of users positively affects their satisfaction.*

*H3b. The degree of the confirmation of users positively affects perceived usefulness.*

*H3c. The degree of the confirmation of users positively affects flow experience.*

### Flow Theory

Flow theory was proposed by Csikszentmihalyi (1975) to explain why people feel happy. Flow is an enjoyable experience that occurs when people are completely immersed in activities and enjoy the goal-achievement process (Csikszentmihalyi, 1990). Having clear goals is a precondition for flow (Csikszentmihalyi, 1990; Lee et al., 2020). In learning, the acquisition of knowledge and skills is regarded as a fundamental goal. Several studies (Zhou, 2013; Stavrou et al., 2015; Huang et al., 2017; Lee et al., 2018; Rodríguez-Ardura and Meseguer-Artola, 2019; Yen and Lin, 2020) have found that skills and challenges are critical antecedents in flow theory. Users become bored when their skills exceed the challenge of a task and become anxious when the challenge of a task exceeds their skills. Thus, a state of flow occurs only when the skills of users match the challenge of a task (Stavrou et al., 2015; Huang et al., 2017; Rodríguez-Ardura and Meseguer-Artola, 2019; Yen and Lin, 2020). Consequently, flow experience arises from a balance between the challenges that individuals face and their ability to overcome these challenges. When such a balance exists, individuals have the confidence and ability to overcome the challenge (Csikszentmihalyi, 1990).

This study considered that a skill–challenge balance exists among users of m-learning apps. The study of Lee et al. (2018) noted that the higher the control and competency of users in meeting the challenge of a gaming system are, the more pleasant their experience would be. The work of Huang et al. (2017) claimed that perceived skill and perceived challenge influence flow experience. The use of information technology is often accompanied by a flow experience, which affects user satisfaction and usage behaviors (Lee et al., 2018; Kim and Hall, 2019). Therefore, this study assumed that if users achieve a skill–challenge balance when using an m-learning app, they could experience a feeling of total involvement which could increase their satisfaction level and continued usage behavior. Consequently, the following hypotheses were proposed:

*H4a. Flow experience positively affects continuance intention to use.*

*H4b. Flow experience positively affects user satisfaction.*

*H5. Perceived skill positively affects flow experience.*

*H6. Perceived challenge positively affects flow experience.*

### Habit

Some studies (Lin et al., 2017; Nascimento et al., 2018; Bolen, 2020) have defined “habits” as automatic behavioral tendencies that individuals develop. Lin et al. (2017) stated that people perform certain behaviors unconsciously because of repeated learning. Thus, people usually engage in certain behaviors continually in a familiar manner without thinking about their behaviors. When used as a moderating variable for the use of information systems, habit limits the effects of other factors (e.g., values, satisfaction, and trust) on the behavioral intentions of users (Hsu et al., 2015; Lin et al., 2017; Nascimento et al., 2018). Thus, habit influences the usage of information systems. Users with stronger habits are less willing to consider the use of new systems (Lin et al., 2017; Nascimento et al., 2018) and thus continue using familiar systems. Based on different perspectives proposed by researchers, this study assumed that perceived usefulness, flow experience, and satisfaction could have varying effects on the continuance intention of learners to use m-learning apps because of the influence of habit. Therefore, the following hypotheses were proposed:

*H7a. The relationship between perceived usefulness and continuance intention to use is moderated by habit.*

*H7b. The relationship between user satisfaction and continuance intention to use is moderated by habit.*

*H7c. The relationship between flow experience and continuance intention to use is moderated by habit.*

## Method

### Instrument Development

The research model comprises seven variables. The items used in this study were slightly modified for the context of m-learning apps. The items to evaluate continuance intention to use were modified versions of the items of Wang et al. (2019), and the habit-related items were adopted from Hsu et al. (2015). Confirmation and user satisfaction were assessed using the scale proposed by Hsu and Lin (2015). The items measuring perceived usefulness and perceived skill were modified versions of the items of Lu et al. (2009) and Ooi et al. (2018). The items for the perceived challenge and flow experience were modified versions of the items of Lee et al. (2018). A 5-point Likert scale ranging from 1 for *strongly disagree* to 5 for *strongly agree* was used to evaluate the constructs. Because the questionnaire was in Chinese, back-translation was conducted. Two bilingual (English and Chinese) experts were invited to translate the original English items used in various studies into Chinese to maintain translation equivalence. To ensure item validity, four professors were asked to comment on the length of the instrument, the format of the instrument, and the wording of the scales. Based on their feedback, some items were modified to suit the research purpose. The results of a pretest indicated the validity of the scale items.

### Data Collection and Sample

To examine the moderating role of habit in continuance usage of m-learning apps, an empirical study was conducted using an online questionnaire survey. The online questionnaires were administered to users of m-learning apps in Taiwan. Invitation messages were posted on popular m-learning forums over an 8-week period. All the respondents were entered into a raffle to win 1 of 25 $5 gift cards. To avoid multiple entries, the identities of the respondents were confirmed through their e-mails and IP addresses after the questionnaires were received. A total of 34 questionnaires, which were duplicates or contained incomplete responses, were eliminated, and 229 valid questionnaires were used for analysis. The response rate was 87.1%. Among the respondents, 52.8% were women, 87.3% were younger than 36 years old, and 66.8% had obtained an undergraduate degree. Most of the m-learning apps used by the respondents were language learning tools.

## Results

### Measurement Model Testing

The measurement model was assessed in terms of the reliability and convergent validity of its constructs. Reliability was determined using Cronbach’s alpha and the composite reliability (CR) model to assess the internal consistency of the measurement model. The Cronbach’s alpha and CR values ranged from 0.86 to 0.93, which exceeded the recommended level of 0.7 (Nunnally, 1978; Fornell and Larcker, 1981). Three measures, namely indicator factor loadings, CR, and average variance extracted (AVE), were used to assess the convergent validity of the instrument (Bagozzi and Yi, 1988). The indicator factor loadings of all items must be higher than 0.5; the CR values should be above0.7; the AVE of each construct must be 0.5 or higher. The factor loading of each item in the research model exceeded0.7; all the CR values were above0.8; the AVE ranged from0.67 to 0.82. These results indicated that all the constructs in the measurement model had acceptable reliability and convergent validity.

Discriminant validity was evaluated using the criterion recommended by Fornell and Larcker (1981), according to which the square root of the AVE for a construct should exceed the correlation coefficients between this construct and all the other constructs in the model. Table 1 indicates that the square root of the AVE for each construct was greater than its correlation with the other constructs, which indicated satisfactory discriminant validity. Thus, the measurement model used in this study had satisfactory reliability, convergent validity, and discriminant validity.

**TABLE 1:** Correlations and average variance extracted (AVE).

Construct	AVE	CF	PU	US	PS	PC	FE	HB	IR
Confirmation (CF)	0.82	**0.91**							
Perceived usefulness (PU)	0.75	0.29	**0.87**						
User satisfaction (US)	0.76	0.37	0.43	**0.87**					
Perceived skill (PS)	0.78	0.27	0.32	0.34	**0.88**				
Perceived challenge (PC)	0.67	0.27	0.36	0.37	0.31	**0.82**			
Flow experience (FE)	0.74	0.31	0.37	0.44	0.40	0.47	**0.86**		
Habit (HB)	0.79	0.25	0.30	0.22	0.22	0.20	0.39	**0.89**	
Continuance intention to use (CU)	0.76	0.38	0.52	0.64	0.27	0.41	0.47	0.32	**0.87**

*The diagonal elements (bold) represent the square roots of the AVE values for the constructs. The off-diagonal elements represent the correlations between the constructs. For discriminant validity, the diagonal elements should be larger than the off-diagonal elements. All the correlations were significant at *p* < 0.01.*

### Structural Model Testing

**FIGURE 2:**
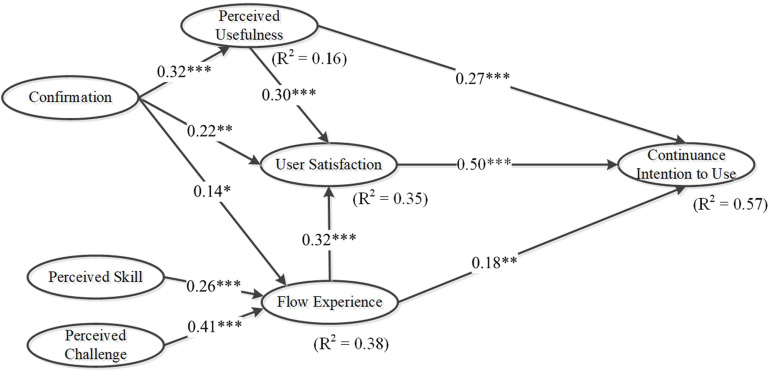
Results of the full sample.

This study used AMOS version 21 to analyze the structural model (Arbuckle, 2012). The structural model exhibited a satisfactory fit with the collected data (χ^2^/*df* = 1.45, goodness of fit index [GFI] = 0.9, adjusted GFI = 0.87, normed fit index = 0.93, comparative fit index = 0.98, root mean square error of approximation = 0.044). Figure 2 presents the standardized coefficients paths, path significance, and variance explained (*R*^2^) by each path. The results indicate that continuance intention to use m-learning apps was predominantly influenced by user satisfaction (β = 0.5, *p* < 0.001), perceived usefulness (β = 0.27, *p* < 0.001), and flow experience (β = 0.18, *p* < 0.01); thus, H1, H2a, and H4a were supported. Similarly, user satisfaction was positively influenced by perceived usefulness (β = 0.3, *p* < 0.001), confirmation (β = 0.22, *p* < 0.01), and flow experience (β = 0.32, *p* < 0.001); therefore, H2b, H3a, and H4b were fully supported. The results also indicated that perceived usefulness was significantly affected by confirmation (β = 0.32, *p* < 0.001); thus, H3b was supported. Finally, confirmation (β = 0.14, *p* < 0.05), perceived skill (β = 0.26, *p* < 0.001), and perceived challenge (β = 0.41, *p* < 0.001) significantly affected flow experience; thus, H3c, H5, and H6 were supported. As illustrated in Figure 2, all the paths were significant at the *p* < 0.05 level or above; thus, H1 to H6 were supported. The variances explained (R^2^) of continuance intention to use m-learning app was 57%, that of user satisfaction was 35%, that of perceived usefulness was 16%, and that of flow experience was 38%.

### Moderating Effect of Habit

To investigate the moderating effect of habit on continuance intention to use, the k-means cluster analysis by Hair et al. (1998) was employed to divide the respondents into the high-habit (*N* = 127) and low-habit (*N* = 102) subgroups. The methods proposed by Yoo (2002) were then used in the AMOS program to examine the differences in path coefficients between these groups in different models. As expected, significant chi-square differences were found between the two groups for Model 1 (χ^2^ = 487.375), Model 2 (γhigh PU-CU = γlow PU-CU, Δχ^2^ = 10.123, Δ*df* = 1, *p* < 0.05), Model 3 (γhigh US-CU = γlow US-CU, Δχ^2^ = 4.092, Δ*df* = 1, *p* < 0.05), and Model 4(γhigh FE-CU = γlow FE-CU, Δχ^2^ = 4.286, Δ*df* = 1, *p* < 0.05), which indicated that habits significantly moderated the paths of perceived usefulness → continuance intention to use, user satisfaction → continuance intention to use, and flow experience → continuance intention to use; thus, H7a, H7b, and H7c were supported. As presented in Table 2, the path coefficients of the low-habit group were lower than those of the high-habit group for the paths of user satisfaction → continuance intention to use and flow experience → continuance intention to use. However, the path coefficient of the low-habit group was higher than that of the high-habit group for the path of perceived usefulness → continuance intention to use.

**TABLE 2:** Results of path coefficients for habit-subgroup.

Hypothesis	Low-habit subgroup		High-habit subgroup	
	β	*t*-statistics	β	*t*-statistics
H1: US → CU	0.28*	2.48	0.51***	5.50
H2a: PU → CU	0.60***	4.95	0.17*	2.20
H2b: PU → US	0.43***	3.88	0.25**	2.80
H3a: CF → US	0.26*	2.56	0.19*	2.05
H3b: CF → PU	0.32**	2.84	0.24*	2.44
H3c: CF → PE	0.13^ns^	1.24	0.14^ns^	1.51
H4a: FE → CU	0.05^ns^	0.58	0.26**	3.02
H4b: FE → US	0.20*	2.01	0.32***	3.92
H5: PS → FE	0.25*	2.25	0.18*	2.02
H6: PC → FE	0.28*	2.34	0.50***	5.07

***p < 0.001, **p < 0.01, *p < 0.05.

*ns(, not significant; US, (user satisfaction; CU, (continuance intention to use; PU, (perceived usefulness; CF(, confirmation; PS, (perceived skill; PC, (perceived challenge; FE, flow experience(.*

## Discussion

This study explored the intention to continue using m-learning apps by using an integrated research model based on ECT, flow theory, and habit. Figure 2 displays the research results of full samples. The findings obtained for intention to continue usage are consistent with those obtained in other studies (Cheng and Yuen, 2018; Li and Fang, 2019; Alshurideh et al., 2020) that indicate that user behavioral intention is positively affected by the satisfaction of users with information technology. In addition, the results of this study suggest that if users perceive that their learning efficiency has increased through the use of an m-learning app, the perceived usefulness of the app would increase, which would affect user behavior. The findings of this study also echoed those of studies that have suggested that flow experience plays a critical role in learning with information systems (Fang et al., 2019; Rodríguez-Ardura and Meseguer-Artola, 2019; Yen and Lin, 2020). If developers improve the design of their m-learning apps to maximize enjoyability, the apps could generate positive feels for users, which may encourage them to continue using the apps.

The results of this study also revealed that the assessment of users of m-learning apps by directly comparing pre-use expectation and post-use performance affects perceived usefulness, which influences user satisfaction. In addition, the experience of the flow of users when using an m-learning app would positively affect their satisfaction. As reported in many studies, the use of information technology is usually accompanied by a flow experience, which affects user satisfaction and usage behavior (Lee et al., 2018; Kim and Hall, 2019). Thus, when users achieve a state of flow in an activity, they become satisfied because their hedonic needs are met.

A central issue in this study was the extent to which other variables influence flow experience. The results indicate that expectation confirmation, perceived skill, and perceived challenge positively affect flow experience, among which perceived skill and perceived challenge are the predominant factors inducing flow experience. Flow experience originates from a balance between challenges and capability. When individuals overcome challenges, their flow experience is positively affected (Zhou, 2013; Huang et al., 2017; Lee et al., 2018; Rodríguez-Ardura and Meseguer-Artola, 2019; Yen and Lin, 2020). Accordingly, a feeling of flow emerges when users are sufficiently skilled and can fulfill the challenge of using teaching content during the learning process.

The results of this study also suggested that habit moderated the relationships of perceived usefulness, satisfaction, and flow experience with continuance intention to use m-learning apps. First, the influence of habit on perceived usefulness was higher for the low-habit users than for the high-habit users. This finding indicated that low-habit users are more aware of the usefulness of m-learning apps (e.g., convenience and learning efficiency) than high-habit ones. Therefore, continuance intention to use m-learning apps was stronger among the low-habit users than among the high-habit users. In addition, the influences of habit on user satisfaction and flow experience were stronger for the high-habit users than for the low-habit users. Compared with low-habit users, high-habit users were more concerned about whether systems could provide satisfaction and flow experience because of their strong habit of using the systems. Overall, the results of this study indicated that habit exerted a moderating effect on the continuance intention of users to use the m-learning apps.

## Implications

The results of this study had several academic and practical implications related to the use of m-learning apps. First, studies have adopted ECT to investigate only the effect of m-learning systems on user behaviors (Lin and Wang, 2012; Dağhan and Akkoyunlu, 2016); however, researchers have recommended that in the context of using information systems for learning, it is necessary to examine the influence on continuance usage intention from the hedonic perspective apart from verifying whether the use of systems can bring about benefits (Cheng and Yuen, 2018; Fang et al., 2019; Rodríguez-Ardura and Meseguer-Artola, 2019; Yen and Lin, 2020). Moreover, when used as a moderating variable for the use of information systems, habit influences user behavioral intention (Hsu et al., 2015; Lin et al., 2017). Therefore, this study considered flow experience and habit to accurately determine the continuance usage intention of users for m-learning systems.

Second, in the context of learning-related information systems, ECT factors (confirmation, perceived usefulness, and user satisfaction), flow factors (perceived skill and perceived challenge), and habit had critical effects on the intention of users to continue using m-learning apps. Moreover, the extent of confirmation and degree of perceived usefulness are both the main antecedents affecting user satisfaction with m-learning apps. These findings provided crucial information for mobile app service providers and indicate that in the context of learning-related information systems, users have high satisfaction when their use experience exceeds their expectations before use. Furthermore, user perceptions of the usefulness of m-learning apps are enhanced if they perceive that the apps are convenient, increase their learning efficiency, and enable them to acquire knowledge and learning opportunities. To retain users, developers of digital teaching materials should focus on the perceptions of users when it comes to the usefulness during the use of m-learning systems. The higher the perception of usefulness among users is, the higher their satisfaction is. High satisfaction may cause users to continue to use a system or service for learning, which would give service providers opportunities to earn a high profit.

Third, this study validated the perspective of Fang et al. (2019) that flow experience was a crucial factor affecting user satisfaction. App designers should consider the joy of learning when designing materials for m-learning apps so that users could experience happiness during the learning process. Such a feeling might enhance the intention of users to continue using m-learning apps.

Fourth, skills and challenges were two key factors in flow theory. Flow occurs only when the skills of users match the challenges they face (Stavrou et al., 2015; Rodríguez-Ardura and Meseguer-Artola, 2019; Yen and Lin, 2020). System designers should understand that users could achieve high learning efficiency when an online learning system has clear goals and can increase their learning motivation. The challenges in m-learning apps could be designed to intrigue learners and increase motivation (positive feelings), which in turn could drive their usage behaviors.

Finally, numerous researchers (Lin et al., 2017; Nascimento et al., 2018) have argued that habit has a moderating influence on the relationships between various factors and the use of information systems. The results of this study indicated that satisfaction has a crucial influence on the intention to continue using a system among low-habit and high-habit users. Moreover, perceived usefulness and flow experience positively affected the intention to continue using m-learning apps among low-habit and high-habit users, respectively. These results were crucial for m-learning system providers and suggested that different levels of habit could have different effects on the intention to use information systems for learning.

## Research Limitations

This study has several limitations. First, the data were collected from users of m-learning apps in Taiwan. The respondents were Chinese-speaking learners of English. Future research should examine and compare users from different countries to test the generalizability of the findings of this study. Second, the cross-sectional investigation method adopted in this study could only be used to examine and explain the situations of participants at a certain time; thus, the investigation was limited to the conditions of a certain period. Consequently, researchers should conduct longitudinal investigations to identify possible changes in perception with experience. Third, this study adopted an online questionnaire and a quantitative statistical research model. The online survey was advertised on several popular websites. To prevent self-selection bias in online surveys, subsequent studies could use an experimental method to support research results. Third, this study adopted an online questionnaire and a quantitative statistical research model.

## Data Availability Statement

The original contributions presented in the study are included in the article/Supplementary Material, further inquiries can be directed to the corresponding author.

## Ethics Statement

The studies involving human participants were reviewed and approved by the Ling Tung University. The patients/participants provided their written informed consent to participate in this study.

## Author Contributions

All authors listed have made a substantial, direct and intellectual contribution to the work, and approved it for publication.

## Conflict of Interest

The authors declare that the research was conducted in the absence of any commercial or financial relationships that could be construed as a potential conflict of interest.

## Publisher’s Note

All claims expressed in this article are solely those of the authors and do not necessarily represent those of their affiliated organizations, or those of the publisher, the editors and the reviewers. Any product that may be evaluated in this article, or claim that may be made by its manufacturer, is not guaranteed or endorsed by the publisher.
