# Supplementary material for: Understanding Continuance Usage of Mobile Learning Applications: The Moderating Role of Habit
Source: Front Psychol. 2021 Nov 10;12:736051. doi: 10.3389/fpsyg.2021.736051 (PMC8631194; doi:10.3389/fpsyg.2021.736051)
Supplement: Supplementary file 1 [file Data_Sheet_1.docx]

**Appendix A. Survey Items**

*Confirmation (CF)*

1. My experience using the m-learning app was better than I expected.
2. The service level or function provided by the m-learning app was better than I expected.
3. Overall, most of my expectations from using the m-learning app were met.

*Perceived usefulness (PU)*

1. Using the m-learning app made it convenient for me to learn.
2. The m-learning app improved my learning efficiency.
3. The m-learning app increased my chances of obtaining additional knowledge.

*User satisfaction (US)*

1. Using the m-learning app made me feel highly satisfied.
2. Using the m-learning app made me feel happy.
3. Using the m-learning app was a positive experience.

*Perceived skill (PS)*

1. Using the m-learning app is entirely within my control.
2. I have the knowledge and ability to use the m-learning app.
3. I am able to skillfully use the m-learning app.

*Perceived challenge (PC)*

1. Using the m-learning app challenges me.
2. Using the m-learning app challenges me to the best of my ability.
3. Using the m-learning app allowed me to test my skills.

*Flow experience (FE)*

1. When I was using the m-learning app, I felt completely engaged.
2. When I was using the m-learning app, time seemed to pass quickly.
3. When I was using the m-learning app, nothing else mattered.

*Habit (HB)*

1. Learning with the m-learning app is something that I do frequently.
2. Learning with the m-learning app is natural for me.
3. Learning with the m-learning app is something that I do without thinking.

*Continuance intention to use (CU)*

1. Assuming that I have access to the m-learning app, I intend to use it again.
2. I will continue using the m-learning app.
3. I will frequently continue using the m-learning app in the future.
